# Effects of short- and long-term mutant IDH1 inhibition on radiosensitivity across genetically diverse patient-derived IDH1-mutant glioma cells

**DOI:** 10.1093/noajnl/vdag057

**Published:** 2026-03-02

**Authors:** Yosuke Kitagawa, Logan D Muzyka, Ami Kobayashi, Ethan Wetzel, Ali Nasser, Julie J Miller, Hiroaki Wakimoto, Daniel P Cahill

**Affiliations:** Translational Neuro-Oncology Laboratory, Massachusetts General Hospital, Harvard Medical School, Boston, MA, 02114, USA; Department of Neurosurgery, Massachusetts General Hospital, Harvard Medical School, Boston, MA, 02114, USA; Translational Neuro-Oncology Laboratory, Massachusetts General Hospital, Harvard Medical School, Boston, MA, 02114, USA; Department of Neurosurgery, Massachusetts General Hospital, Harvard Medical School, Boston, MA, 02114, USA; Department of Neurology, Brigham and Women’s Hospital, Harvard Medical School, Boston, MA, 02115, USA; Translational Neuro-Oncology Laboratory, Massachusetts General Hospital, Harvard Medical School, Boston, MA, 02114, USA; Department of Neurosurgery, Massachusetts General Hospital, Harvard Medical School, Boston, MA, 02114, USA; Translational Neuro-Oncology Laboratory, Massachusetts General Hospital, Harvard Medical School, Boston, MA, 02114, USA; Department of Neurosurgery, Massachusetts General Hospital, Harvard Medical School, Boston, MA, 02114, USA; Translational Neuro-Oncology Laboratory, Massachusetts General Hospital, Harvard Medical School, Boston, MA, 02114, USA; Pappas Center for Neuro-Oncology, Department of Neurology, Massachusetts General Hospital, Harvard Medical School, Boston, MA, 02114, USA; Translational Neuro-Oncology Laboratory, Massachusetts General Hospital, Harvard Medical School, Boston, MA, 02114, USA; Department of Neurosurgery, Massachusetts General Hospital, Harvard Medical School, Boston, MA, 02114, USA; Translational Neuro-Oncology Laboratory, Massachusetts General Hospital, Harvard Medical School, Boston, MA, 02114, USA; Department of Neurosurgery, Massachusetts General Hospital, Harvard Medical School, Boston, MA, 02114, USA

**Keywords:** cytotoxicity, glioma, IDH inhibitor, radiation therapy, 2-hydroxyglutarate

## Abstract

**Background:**

IDH mutant gliomas produce the oncometabolite 2-hydroxyglutarate (2-HG), driving tumorigenesis through metabolic dysregulation and epigenetic alterations. IDH inhibitors (IDHi) reduce 2-HG and are clinically approved for treating IDH mutant gliomas. However, the observed impact of IDHi therapy on tumor response to ionizing radiation (IR) has been variable across murine models and engineered cell lines.

**Methods:**

We investigated the effects of short-term (5 days) and long-term (≥5 weeks) exposure to the IDH1 inhibitor AGI-5198 on radiation-induced cytotoxicity. Patient-derived glioma neurosphere lines (MGG119, TS603S2, BT142, and MGG152) were studied, with IDH1-mutant fibrosarcoma HT1080 and an inducible IDH1-R132H glioma line (MGG18 Tet±). Intracellular 2-HG, cell viability, and clonogenic survival were measured following IR.

**Results:**

AGI-5198 potently reduced intracellular 2-HG across all IDH1-mutant lines after short-term treatment, with suppression maintained during prolonged exposure but rapidly reversed upon withdrawal. Long-term AGI exposure produced cell viability responses to both standard- and high-dose IR comparable to short-term treatment in HT1080, TS603S2, BT142, MGG152, and MGG18 Tet±. Across endogenous IDH-mutant models, neither short- nor long-term IDH inhibition induced radioresistance. MGG119, harboring IDH1-R132H and MET alterations, showed intrinsic radioresistance unaffected by IDHi. In contrast, MGG152, harboring IDH1-R132H and BRCA2 mutations, exhibited modest radiosensitization with IDHi.

**Conclusions:**

Prolonged AGI-5198 exposure does not reduce IR sensitivity in IDH mutant glioma cells. Effects were comparable to short-term treatment, while radiation responses varied by genetic context. No deleterious interaction between IDHi and IR was observed in endogenous IDH-mutant cells except for MGG18 Tet+ supporting integration of IDHi with radiotherapy in IDH mutant gliomas.

Key Points

- Long-term IDH1 inhibition did not induce radioresistance in glioma models
- Radiosensitization was observed only in glioma cells with BRCA2 mutation
- 2-HG suppression was reversible within 1-2 weeks after drug withdrawal

Importance of the Study

The clinical approval of IDH inhibitors introduces a need to define their interaction with radiotherapy, a standard treatment for glioma. Using patient-derived IDH1-mutant glioma models, an inducible system, and IDH1-mutant fibrosarcoma, we compared short- versus long-term AGI-5198 exposure in combination with ionizing radiation. We found that AGI-5198 consistently suppressed 2-hydroxyglutarate, but this effect was rapidly reversible after withdrawal. Importantly, prolonged exposure did not confer radioresistance; responses were comparable to short-term treatment across most models. Genetic context shaped outcomes: MGG119 with a MET alteration remained intrinsically resistant, while MGG152 with a BRCA2 mutation showed modest radiosensitization. These findings indicate that long-term IDH inhibition does not compromise the efficacy of radiation and support the safe integration of IDH inhibitors with radiotherapy. The data also highlight the importance of tumor-specific genomic background in determining therapeutic response.

Gliomas represent the most common form of primary malignant brain tumors in adults and remain a clinical management challenge in neuro-oncology. The incorporation of molecular diagnostics, particularly the identification of mutations in isocitrate dehydrogenase (IDH), has significantly refined the classification, prognostication, and therapeutic targeting of gliomas.^1–3^ Mutations in the gene encoding *IDH1* are found in approximately 20-25% of all diffuse gliomas.^4^^,^^5^ These mutations not only serve as molecular hallmarks but also play a central role in glioma pathogenesis, highlighting the importance of molecular profiling for guiding therapeutic decisions and informing prognosis.^6^^,^^7^

*IDH* mutations lead to the aberrant production of D-2-hydroxyglutarate (2-HG), an oncometabolite, that disrupts normal cellular metabolism and epigenetic regulation, thereby promoting tumorigenesis.^8–12^ This metabolic activity establishes a critical mechanistic link between metabolic dysregulation and glioma progression.^13^ Despite advances in characterizing the molecular landscape of IDH-mutant gliomas, the specific roles of 2-HG *in* *vivo* versus *in* *vitro* remain incompletely understood. Moreover, its precise contribution to tumor maintenance and therapeutic response continues to be actively investigated.^14^^,^^15^

Recent advancements in targeting mutant IDH enzymes in gliomas have led to the development of specific inhibitors, such as ivosidenib (AG-120) and vorasidenib (AG-881), which have demonstrated effectiveness in clinical trials.^16^^,^^17^ These agents reduce the accumulation of the oncometabolite 2-HG and modulate the tumor microenvironment, highlighting their therapeutic potential. AGI-5198, a selective IDH1-R132H inhibitor, has been shown to reduce tumor growth *in* *vivo*, and promote differentiation in IDH1-mutant glioma cells.^18^ Most notably, a recent phase 3 clinical trial demonstrated that vorasidenib significantly improved progression-free survival in patients with grade 2 IDH-mutant gliomas, leading to FDA approval.^19^ With the addition of a new therapeutic option for patients, critical uncertainties remain regarding how best to integrate these agents with other treatment modalities, particularly ionizing radiation therapy (IR), which remains a cornerstone of standard-of-care glioma management. Early mechanistic studies suggested that *IDH1* mutations or 2-HG production increase radiosensitivity by enhancing oxidative stress and impairing DNA repair, ultimately promoting apoptosis following irradiation.^20–23^ These findings offered a mechanistic rationale for the therapeutic window and improved clinical outcomes observed in patients with IDH-mutant gliomas undergoing radiotherapy. Indeed, Molenaar et al. reported that AGI-5198 reversed radiosensitization and protected IDH1-mutant cells from IR-induced damage,^24^ raising the possibility that application of IDH inhibitor at the same time as radiation treatment might narrow the therapeutic window and blunt the clinical effectiveness of this established treatment modality. In other studies, however, Kadiyala et al. reported that pharmacologic inhibition of 2-HG production by AGI-5198 sensitized IDH1-mutant gliomas to radiotherapy and prolonged survival in preclinical models.^25^

Taken together, these opposing findings highlight the need for further investigation into the interplay between IDH inhibition and radiotherapy. Critically, it remains unclear whether IDH mutant glioma cells subjected to long-term pharmacological IDH inhibiton retain sensitivity to delayed radiation exposure, as might occur in real-world clinical settings where patients receive IDH inhibitors prior to radiotherapy.

In this study, we sought to provide evidence addressing this unresolved question by evaluating how short-term versus long-term exposure to AGI-5198 affects radiation-induced cytotoxicity in IDH1-mutant glioma cells. Using a panel of patient-derived cell lines and an IDH1-mutated fibrosarcoma line, we assess the kinetics of 2-HG suppression and cellular viability following IR. This work aims to provide insights into the appropriate integration of IDH inhibitors into multimodal treatment approaches.

## Materials and Methods

### Cell Lines

The patient-derived glioma cell lines used in this study; MGG18 (IDH-wildtype), MGG119 (IDH1-R132H), and MGG152 (IDH1-R132H), were obtained between 2008 and 2013 under IRB-approved protocols.^26^ The TS603 gliomasphere line, derived from a WHO grade III anaplastic oligodendroglioma patient with *IDH1*-R132H mutation, was obtained from Memorial Sloan-Kettering Cancer Center. TS603S2 is a subclone of TS603 cells isolated and selected based on stable expression of IDH1-R132H. BT142 was obtained from ATCC. The human fibrosarcoma cell line HT1080 (IDH1R132C) was obtained from ATCC and authenticated by STR profiling to match the ATCC dataset. The patient-derived glioma neurosphere lines (TS603S*2*, MGG18, MGG119, and MGG152) were cultured in serum-free Neurobasal medium (Gibco) supplemented with N2, B27, EGF and FGF2, following the established protocol.^27–29^ HT1080 cells were maintained in EMEM, supplemented with 10% fetal bovine serum and antibiotics, including penicillin, streptomycin and amphotericin B. All cell lines were confirmed to be mycoplasma-free using the LookOut Mycoplasma PCR Detection Kit (Sigma) and were cultured at 37°C in a humidified atmosphere of 5% CO_2_/95% air.

### Inducible IDH1-R132H Cell Line Generation

MGG18-IDH1-R132H cells, expressing tetracycline-inducible *IDH1*-R132H mutation, were generated through lentiviral transduction as previously described.^14^ For mutant IDH1 expression induction, MGG18-IDH1-R132H cells were treated with 1 μg/mL doxycycline (Sigma-Aldrich) for greater than 3 months.

### Compounds and Chemicals

The compounds used in this study included AGI-5198 (MedChemExpress, HY-18082) and dimethyl sulfoxide (DMSO; Sigma-Aldrich), which were added to the cell culture media where indicated.

### Irradiation

For the irradiation experiments, cells were exposed to ionizing radiation using an X-RAD 320 X-ray irradiator (Precision X-Ray, North Branford, CT) operated at 320 kVp and 12.5 mA with a 2.0-mm aluminum filter at a source-to-sample distance of 50 cm. Under these conditions, the dose rate was approximately 2.3-2.4 Gy/min. Cells were irradiated at room temperature in white 96-well plates with the lids on, with each well containing 100 μL of culture medium.

### Cell Viability and Clonogenic Assays

Cells exposed to DMSO or AGI-5198 (5 days and 5 weeks or longer) were seeded in 96-well plates at densities ranging from 1,000 to 3,000 cells per well and irradiated 6 h after plating. IDH inhibitor treatment was maintained without washout throughout the post-irradiation period. After irradiation, cells were cultured for 72 h in HT1080 cells and for 96 to 120 h in glioma neurosphere cells under suspension conditions, depending on the growth characteristics of each line, before assessment of cell viability using the CellTiter-Glo assay (CTGA, Promega). Viability results were presented as a percentage relative to the DMSO control. For clonogenic assay, HT1080 cells, treated with either DMSO or AGI-5198 (5 μmol/L) for specified durations, were seeded at 100 cells per well in 6-well plates, with the same DMSO or AGI-5198 treatment continued. Cells were irradiated 24 h later and incubated for 7 days. Cells were washed, fixed in 6% glutaraldehyde, and stained with 0.5% crystal violet. Colonies were counted under an inverted microscope in a blinded manner by A.N. to eliminate bias.

### 2-HG Quantification

For 2-HG quantification, 1 × 10^6^ cells were lysed using CelLytic M Cell Lysis Reagent (Sigma-Aldrich), centrifuged at 13,000 rpm for 15 min, and treated with 4 M perchloric acid (PCA). Following neutralization with 2 M potassium hydroxide (KOH) and pH adjustment to 6.5-8.0, the samples were incubated with the 2-HG reaction mixture at 37°C for 60 min. Fluorescence was measured using a SpectraMax iD3 Multi-Mode Microplate Reader, with an excitation wavelength of 530 nm and an emission wavelength of 590 nm.

### Western Blot Analysis

Cells were harvested and lysed in radioimmunoprecipitation assay (RIPA) buffer (Thermo Fisher Scientific) supplemented with protease and phosphatase inhibitor cocktails (Roche). Cell lysates containing equal amounts of total protein (10-20 μg) were separated via 4-20% SDS-PAGE and transferred to polyvinylidene difluoride (PVDF) membranes (Bio-Rad). Membranes were blocked with 5% (w/v) non-fat dry milk in TBS-T buffer (20 mM Tris, pH 7.5; 150 mM NaCl; 0.1% Tween 20) for 1 h at room temperature. Membranes were then probed with the following primary antibodies overnight at 4°C: IDH1 R132H (mouse monoclonal antibody; Dianova, Cat# DIA-H09, RRID: AB_2335716; dilution 1:1,000), and Cyclophilin B (rabbit monoclonal antibody; Cell Signaling Technology, Cat# 43603, RRID: AB_2799247; dilution 1:1,000).

After primary antibody incubation, membranes were washed with TBS-T and incubated with horseradish peroxidase (HRP)-linked secondary antibodies for 1 h at room temperature. The secondary antibodies used were: ECL Anti-Mouse IgG HRP-linked whole antibody from sheep (Cytiva, Cat# NA931, RRID: AB_772210; dilution 1:5,000) for the mouse primary antibody (IDH1 R132H), ECL Anti-Rabbit IgG HRP-linked whole antibody from donkey (Cytiva, Cat# NA934, RRID: AB_772206; dilution 1:5,000) for the rabbit primary antibody (Cyclophilin B).

Following final washes with TBS-T, membranes were exposed to ECL substrate (Bio-Rad) for 5 min before visualization using the Gel Doc XRS+ imaging system (Bio-Rad, RRID: SCR_019690). Images were captured using Image Lab Software (Bio-Rad, RRID: SCR_014210), exported as TIFF files at 600 DPI resolution, and cropped for presentation.

### Statistical Analysis

Statistical analyses were performed with GraphPad Prism software v10 (RRID: SCR_002798). Differences were compared using unpaired Student’s t-test for 2 groups or ANOVA for multiple groups, with correction for multiple comparisons. All studies used a cutoff of significance of *P* < .05.

## Results

### 2-HG Suppression is Sustained by Prolonged IDH Inhibitor Treatment with Rapid Reversal upon Discontinuation in IDH1-Mutant Glioma Cells

**Figure 1.**
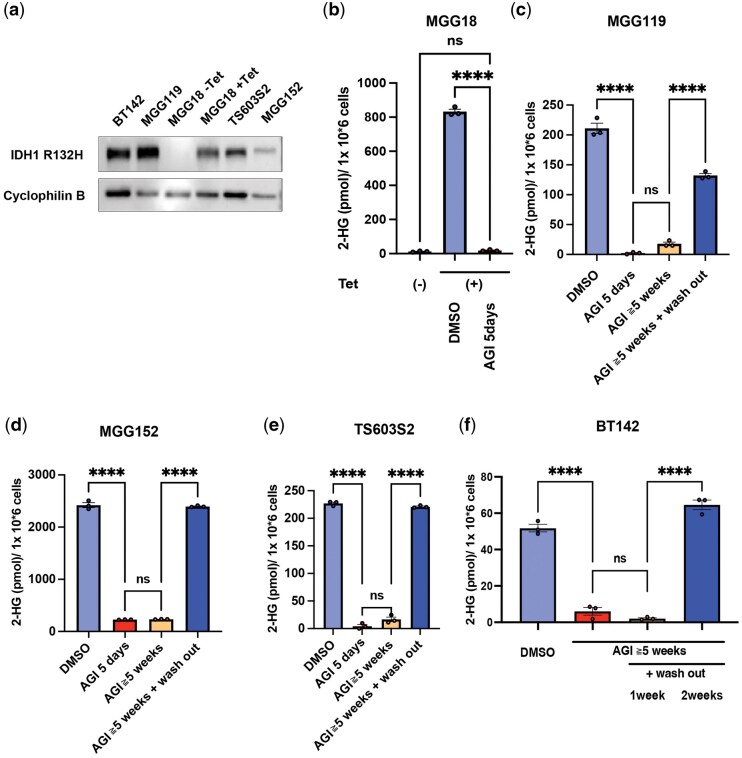
Long-term AGI-5198 treatment maintains 2-HG suppression, which is rapidly reversed upon discontinuation across all IDH1-mutant glioma cells. (a) Western blot analysis of IDH1-R132H expression in glioma cell lines: BT142, MGG119, MGG18 without doxycycline (Tet−), MGG18 with doxycycline (Tet+), TS603S2, and MGG152. MGG18 is a tet-inducible IDH1-R132H model. Cyclophilin B served as a loading control. (b) 2-hydroxyglutarate (2-HG) levels in MGG18 (Tet− and Tet+) after AGI-5198 (AGI) treatment (5 μM) for 5 days. (c-f) 2-hydroxyglutarate (2-HG) levels in (c) MGG119, (d) MGG152, (e) TS603S2, and (f) BT142 after AGI-5198 treatment (5 μM) for 5 days or more than 5 weeks and following a 1- or 2-week drug washout. Data are presented as mean ± SEM. Ns indicates not significant; **** indicates *P* < .0001.

AGI-5198 is a selective, allosteric inhibitor of the mutant IDH1-R132H enzyme, inhibiting the synthesis of 2-HG in both mouse and human glioma cells.^11^^,^^30^^,^^31^ We first sought to understand the impact of duration of AGI-5198 treatment on the persistence of its 2-HG inhibitory effect. To address this, we used multiple glioma cell lines harboring *IDH1*-R132H mutation (Table 1, Figure 1a). In MGG18 cells, IDH1-R132H expression was conditionally induced by doxycycline (Tet), as confirmed by western blot analysis (Figure 1a). Tet induction led to a robust increase in intracellular 2-HG, which was effectively reversed following 5 days of 5µM AGI-5198 treatment, returning to near-baseline levels (Figure 1b). We next assessed the kinetics and durability of 2-HG suppression by measuring intracellular levels over time in endogenous IDH1-R132H-mutant glioma cell lines. In MGG119, MGG152, TS603S2, and BT142 cells, a marked reduction in 2-HG levels was observed after 5 days of treatment and was sustained over a 5-week period of continuous AGI-5198 exposure (Figure 1c-f). Notably, this reduction was rapidly reversed following a 1-week washout period, with 2-HG levels returning to approximately 70-100% of baseline in MGG119, MGG152, and TS603S2 cells (Figure 1c-e). In BT142 cells, 2-HG levels remained suppressed for 1-week post-washout, but returned to near-baseline levels after 2 weeks (Figure 1f). These results indicate that AGI-5198 effectively sustains 2-HG suppression over an extended period across various IDH1-mutant glioma cells, and that levels of 2HG are restored following discontinuation of AGI-5198.

**Table 1.** Cell lines used in this study with genetic background and factors potentially influencing radiosensitivity

Cell lines	IDH1 mutation	Additional genetic background/factor
HT1080 fibrosarcoma cells	IDH1-R132C	CDKN2A deletion
MGG119 cells	IDH1-R132H	ATRX deletion, TP53 mutation, CDKN2A, MET alteration
TS603S2 cells	IDH1-R132H	ATRX, TERT, RB1, TP53 mutation, CDKN2A deletion
BT142 cells	IDH1-R132H	ATRX, TP53 mutation
MGG152 cells	IDH1-R132H	ATRX, TP53, BRCA2 mutation
MGG18 Tet- cells (genetically engineered)	No IDH1 mutation	TERT, RB1, TP53 mutation
MGG18 Tet+ cells (treated with 1 μg/mL doxycycline; genetically engineered)	IDH1-R132H	TERT, RB1, TP53 mutation

### Prolonged AGI Exposure Results in Similar Cell Viability Responses to High-Dose Irradiation as Short-Term AGI Treatment in IDH1-Mutant Fibrosarcoma Cells

**Figure 2.**
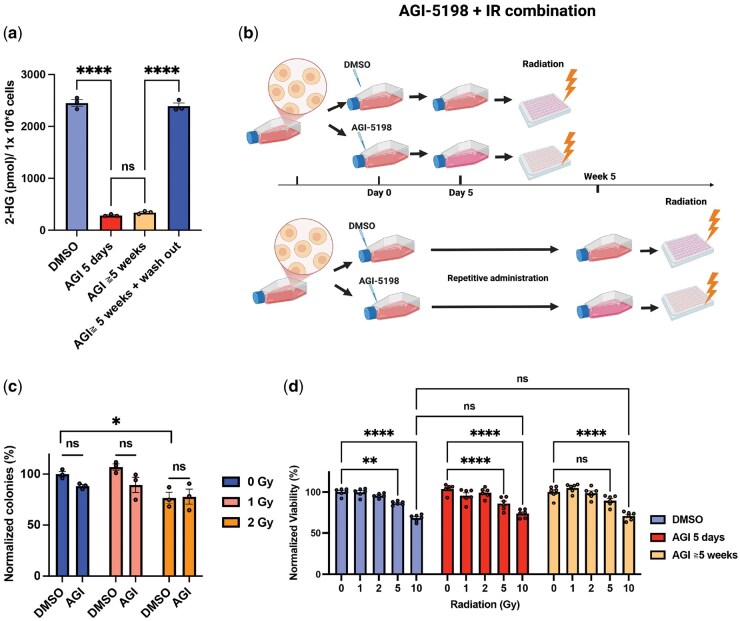
Short- and long-term AGI-5198 treatment does not alter cell viability responses to irradiation in IDH1-mutant fibrosarcoma cells. (a) Intracellular 2-hydroxyglutarate (2-HG) levels measured after 5-day or 5-week AGI-5198 (AGI) treatment (5 μM), and after washout following 5-week treatment in IDH1-mutant HT1080 cells. (b) Workflow of the AGI-5198 treatment and radiation experiment (created with BioRender.com) (c) Clonogenic assay of HT1080 cells treated with DMSO or AGI-5198 (5 μM) for 5 days prior to irradiation (0, 1, or 2 Gy). (d) Cell viability at 72 h after irradiation (0-10 Gy) following 5-day or 5-week AGI-5198 treatment (5 μM). Data are shown as mean ± SEM; ns indicates not significant; *P < .05; **P < .01; ****P < .0001.

To investigate how treatment duration may affect cellular responses to IR, we utilized HT1080 fibrosarcoma cells, which harbor *IDH1*-R132C mutation. These cells demonstrated a comparable reduction in 2-HG levels following AGI-5198 treatment as observed in IDH1-R132H-mutant glioma cell lines (Figure 2a). Suppression of 2-HG was evident after 5 days of AGI-5198 treatment and persisted over a 5-week period, with levels rapidly reversing within 1-week of washout (Figure 2a). We next performed both a clonogenic assay and a Cell Titer-Glo (CTG) viability assessment assay on HT1080 cells treated with AGI-5198 in combination with IR (Figure 2b). As expected, reduced colony formation was observed with IR at 2 Gy in control (DMSO-treated) cells. Short-term (5-day) AGI-5198 exposure alone did not alter clonogenicity (Figure 2c). Importantly, short-term AGI-5198 treatment did not enhance or reduce radiation-induced cytotoxicity at 1-2 Gy doses (Figure 2c). CTG assays revealed a dose-dependent reduction in cell viability with IR at doses above 5 Gy, particularly at 10 Gy, for both short-term (5-day) and prolonged (≥5-week) AGI-5198 treatments (Figure 2d). Importantly, IDH inhibition with AGI-5198 did not result in either radioresistance or enhanced radiosensitization at these doses. Thus, neither short-term (5-day) nor prolonged (≥5-week) AGI-5198 treatment alters the radiosensitivity of HT1080 cells in response to standard-dose or high-dose IR.

### Cell Viability Responses to High-Dose Irradiation across a Panel of IDH1-Mutant Glioma Cells

**Figure 3.**
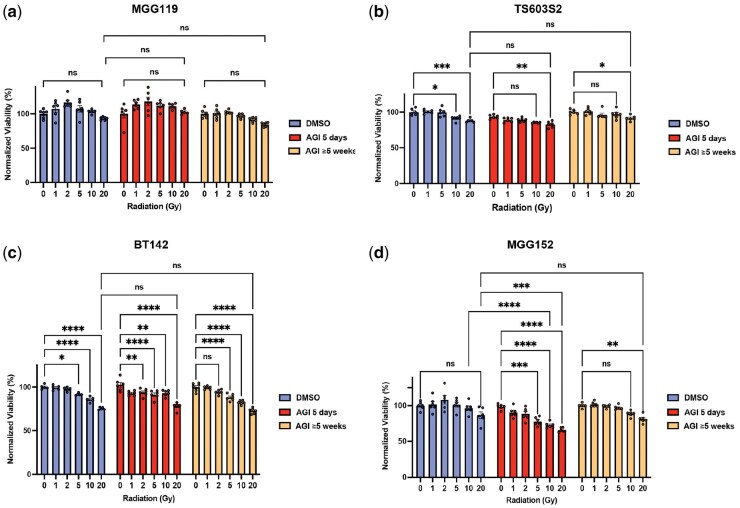
Cell viability responses to irradiation in endogenous IDH1-mutant glioma cells after short- and long-term AGI-5198 treatment. (a-d) Normalized viability of (a) MGG119, (b) TS603S2, (c) BT142, and (d) MGG152 IDH-mutant glioma cells 120 h after exposure to increasing doses of irradiation (0, 1, 2, 5, 10, and 20 Gy) following treatment with DMSO (control), short-term 5 μM AGI-5198 (AGI) (5 days), or long-term 5 μM AGI-5198 (≥5 weeks). Data are presented as mean ± SEM. ns indicates not significant; *P < .05; **P < .01; ***P < .001; ****P < .0001.

To determine whether the effect of AGI-5198 on high-dose radiation-induced cytotoxicity observed in HT1080 cells is recapitulated in human glioma cell lines harboring the *IDH1*-R132H mutation, we performed CTG assays on MGG119, TS603S2, BT142, and MGG152 cells. Consistent with our findings in HT1080 cells, a dose-dependent reduction in cell viability with IR was observed for both short-term (5-day) and prolonged (≥5-week) AGI-5198 treatments in various IDH-1 mutant glioma cells except for MGG119 (Figure 3a-d). MGG119 cells, which carry both the *IDH1*-R132H mutations and *MET* alterations, were intrinsically resistant to IR, and this phenotype was not affected by treatment with AGI-5198 of any duration (Figure 3a). Interestingly, in MGG152 cells—which harbor both the *IDH1*-R132H and *BRCA2* mutations^26^—short-term AGI-5198 exposure displayed small but reproducible enhancement in radiation-induced cytotoxicity at doses ≥10 Gy (Figure 3d). These findings overall verify the lack of major impact of short or long-term AGI-5198 treatment on cellular response to IR. These data also suggest that specific genetic contexts such as MET alteration and BRCA deficiency may differentially modulate the cellular response to AGI-5198-mediated radiosensitization.

### Tetracycline-Induced IDH1-R132H Mutation Modestly Enhances Radiosensitivity with Short-Term AGI Treatment

**Figure 4.**
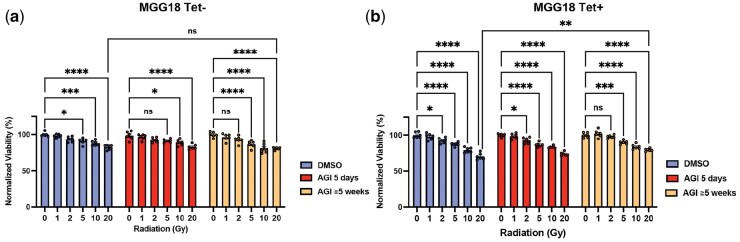
Radiosensitivity and modulation by AGI-5198 in tetracycline-induced IDH1-R132H mutant glioma cells. (a-b) Normalized viability of MGG18 cells (a) without (Tet−) and (b) with doxycycline induction (Tet+) of IDH1-R132H expression, at 120 h after exposure to increasing doses of irradiation (0, 1, 2, 5, 10, and 20 Gy) following treatment with DMSO (control), short-term 5 μM AGI-5198 (AGI) (5 days), or long-term 5 μM AGI-5198 (≥5 weeks). Data are presented as mean ± SEM. ns indicates not significant; *P < .05; **P < .01; ***P < .001; ****P < .0001.

To investigate the role of IDH1-R132H expression in modulating the effects of AGI-5198 on radiation-induced cytotoxicity, we utilized MGG18 cells, originally derived from an IDH-wildtype glioblastoma, engineered with a tet-inducible IDH1-R132H mutant transgene (Tet+/–). In Tet– cells (no IDH1-R132H expression), we observed a dose-dependent reduction in cell viability following IR, with no significant effects of short-term (5-day) and prolonged (≥5-week) AGI-5198 treatments, as expected (Figure 4a). Tet+ cells (with IDH1-R132H expression) exhibited slightly enhanced ­sensitivity to IR compared with Tet– cells, showing a dose-dependent reduction in cell viability at 2 Gy and above, which was not affected by short-term AGI-5198 exposure (Figure 4b). After extended AGI-5198 treatment, cell viability at 20 Gy was modestly higher compared to DMSO controls, suggesting a potential attenuation of radiation-induced cytotoxicity at very high IR doses (Figure 4b). These findings indicate that AGI-5198, under specific conditions, may attenuate the cellular response to irradiation rather than enhancing radiosensitivity, aligning with previous findings.^24^

## Discussion

Previous genetic studies have established mutations in *IDH1* as a key driver of tumorgenesis and a compelling target for therapeutic intervention.^32–36^ Pharmacologic inhibition of mutant IDH1, leading to reduced production of ­oncometabolite 2-HG, has been shown to confer therapeutic benefit.^17^^, ^^37^ In this study, we demonstrate that AGI-5198 effectively reduces intracellular 2-HG levels across all tested cell lines after just 5 days of exposure, with sustained suppression during extended treatment (Figure 1b-f). Importantly, upon drug washout, 2-HG levels returned to baseline within 1-2 weeks, highlighting the reversible nature of AGI-5198’s metabolic effects (Figure 1b-f). These findings underscore the need for continuous IDH1 inhibition to maintain reduced 2-HG levels.

In addition, previous studies have demonstrated that targeted inhibition of mutant *IDH1*, in combination with IR, effectively suppresses the growth of IDH1-R132H mutant glioma neurospheres and tumors.^15^^,^^25^ In our study, long-term exposure to AGI-5198 resulted in similar cell viability response to IR as short-term AGI treatment in various *IDH1*-R132H mutant cell lines (Figures 2d and 3b-d). An instructive exception was the MGG119 cell line, which harbors both the *IDH1*-R132H mutation and *MET* alteration, according to our previous study on genomic profiling of our various IDH-mutant cell lines (Table 1).^26^  *MET* gene, a proto-oncogene encoding a receptor tyrosine kinase (c-MET), also known as the hepatocyte growth factor receptor, plays an essential role in cellular processes such as growth, survival, migration and differentiation. In MGG119 cells, *MET* alterations are characterized by MET fusion where *MET* undergoes fusion with another gene such as *PTPRZ1*.^27^^, ^^38^^, ^^39^ This fusion results in the production of a constitutively activated receptor, which can drive oncogenic signaling pathways and contribute to tumorigenesis. Several studies have shown that MET can contribute to radioresistance of conventional cell lines,^40^^,^^41^ which may explain the persistent radioresistance observed in MGG119 cells despite treatment with AGI-5198, irrespective of the treatment duration (Figure 3a).

Furethermore, MGG152 cell line, which harbors concurrent mutations in *IDH1*-R132H and *BRCA2* (Table 1) exhibited reproducible enhancement in radiation-induced cytotoxicity at doses ≥10 Gy following short-term AGI-5198 exposure (Figure 3d). This selective radiosensitization observed in MGG152 likely reflects a unique vulnerability in DNA repair pathways, specifically involving homologous recombination repair (HRR).^13^ The *BRCA2* mutation in MGG152 may confer HRR deficiency impairing the resolution of DNA double-strand breaks from high-dose radiation.^42^ Thus, in this context, we speculate that short-term AGI-5198-mediated 2-HG depletion may exacerbate radiation-induced DNA damage, unmasking underlying HRR defects and enhancing cytotoxicity.

Notably, this radiosensitizing effect was not maintained during prolonged AGI-5198 exposure, suggesting the activation of compensatory cellular mechanisms that restore DNA repair capacity or engage alternative repair pathways. These findings demonstrate that while IDH1 inhibitors may partially reverse epigenetic dysregulation, chromatin structure, and oxidative stress through 2-HG depletion,^13^ such effects alone were insufficient to sensitize most IDH1-mutant glioma cells to IR. Although 2-HG modulates chromatin structure and the DNA repair machinery, compensatory pathways, such as lysine demethylase activity and alternative DNA repair mechanisms, may mitigate the effects of IDH inhibition.^12^^,^^43^ The observation of radiosensitization in only a single model emphasizes the biological heterogeneity of IDH-mutant gliomas and the importance of genetic context in determining therapeutic response.

These findings have direct relevance to the current clinical management of IDH-mutant glioma, in which mutant IDHis are increasingly chosen post-operatively to defer radiotherapy in select clinical settings, particularly in patients with grade 2 IDH-mutant gliomas.^19^^,^^44^ In this context, lack of deleterious impact of IDHi on response to IR across endogenous IDH-mutant glioma models suggests that IDHi treatment is unlikely to compromise the efficacy of subsequent radiotherapy, supporting the clinical practice and the feasibility of administering radiotherapy after a period of IDH inhibitor treatment. At the same time, our results also indicate that a subset of models exhibit context-dependent alterations in radiation response. These findings highlight that the interaction between IDH pathway blockade and the DNA damage reponse may be influenced by cellular context, such as genetic background and metabolic state, underscoring the need for further research and careful consideration of treatment sequencing.

Several limitations of this study should be acknowledged. First, our studies were focused on *in vitro* experiments using neurosphere models derived from IDH-mutant malignant gliomas, and did not validate findings i*n vivo*. While these neurosphere systems preserve key molecular features of glioma, they do not fully recapitulate the spatial organization, metabolic gradients, and microenvironmental complexity of tumors *in vivo*. In addition, radiation response was assessed under suspension-based neurosphere conditions, where 3-dimensional sphere architecture may influence effective dose distribution and attenuation and radiation reponses. For glioma neurosphere cell lines, cell viability assays were used as the primary readout rather than clonogenic survival assays, which is the gold standard for radiosensitivity assessment. Future work should include quantitative morphologic analyses of neurospheres and clonogenicity measurements such as limiting dilution assay^45^ and neurosphere-adapted clonogenic approaches.^46^ Finally, given the model-specific differences inherent to IDH-mutant glioma systems, the observed effects of combining IDH inhibition with radiotherapy may reflect context-dependent confounders, and therefore IDH1 inhibition may possibly reduce rather than enhance radiosensitivity as we found in Tet-induced IDH1-R132H+ cells.

This study utilized AGI-5198, a selective inhibitor of mutant IDH1, which has been widely used in preclinical research. Both AGI-5198 and the clinically approved IDHi vorasidenib (AG-881) are allosteric inhibitors of mutant IDH1 capable of potent abrogation of 2HG production. It must be noted, however, that they differ in target spectrum and pharmacokinetic properties as vorasidenib covers mutant IDH2, retains activity against wildtype IDH, and is brain-penetrant.^47^^,^^48^ These pharmacologic distinctinons entail that AGI-5198 may not fully recapticulate the clinical effects of vorasidenib. Accordingly, our findings should be cautiously interpreted as mechanistic insight into the interaction between mutant IDH1 inhibition and radiotherapy response rather than a direct prediction of clinical responses.

## Data Availability

All data related to this paper may be requested from the authors.

## References

[1] Louis DN , Perry A, Wesseling P, et al The 2021 WHO classification of tumors of the central nervous system: a summary. Neuro Oncol. 2021;23:1231-1251. 10.1093/neuonc/noab106 34185076 PMC8328013

[2] Weller M , Van Den Bent M, Preusser M, et al EANO guidelines on the diagnosis and treatment of diffuse gliomas of adulthood. Nat Rev Clin Oncol. 2021;18:170-186. 10.1038/s41571-020-00447-z 33293629 PMC7904519

[3] Horbinski C , Berger T, Packer RJ, Wen PY. Clinical implications of the 2021 edition of the WHO classification of central nervous system tumours. Nat Rev Neurol. 2022;18:515-529. 10.1038/s41582-022-00679-w 35729337

[4] Robinson C , Kleinschmidt-DeMasters BK. *IDH1* -Mutation in diffuse gliomas in persons age 55 years and over. J Neuropathol Exp Neurol. 2017;76:151-154. 10.1093/jnen/nlw112 28110298

[5] Iorgulescu JB , Torre M, Harary M, et al The misclassification of diffuse gliomas: rates and outcomes. Clin Cancer Res. 2019;25:2656-2663. 10.1158/1078-0432.CCR-18-3101 30635340 PMC6467794

[6] Miller JJ , Gonzalez Castro LN, McBrayer S, et al Isocitrate dehydrogenase (IDH) mutant gliomas: a society for neuro-oncology (SNO) consensus review on diagnosis, management, and future directions. Neuro Oncol. 2023;25:4-25. 10.1093/neuonc/noac207 36239925 PMC9825337

[7] Ostrom QT , Price M, Neff C, et al CBTRUS statistical report: primary brain and other central nervous system tumors diagnosed in the United States in 2015–2019. Neuro Oncol. 2022;24:v1-v95. 10.1093/neuonc/noac202 36196752 PMC9533228

[8] Dang L , White DW, Gross S, et al Cancer-associated IDH1 mutations produce 2-hydroxyglutarate. Nature. 2009;462:739-744. 10.1038/nature08617 19935646 PMC2818760

[9] Xu W , Yang H, Liu Y, et al Oncometabolite 2-Hydroxyglutarate is a competitive inhibitor of α-ketoglutarate-dependent dioxygenases. Cancer Cell. 2011;19:17-30. 10.1016/j.ccr.2010.12.014 21251613 PMC3229304

[10] Figueroa ME , Abdel-Wahab O, Lu C, et al Leukemic IDH1 and IDH2 mutations result in a hypermethylation phenotype, disrupt TET2 function, and impair hematopoietic differentiation. Cancer Cell. 2010;18:553-567. 10.1016/j.ccr.2010.11.015 21130701 PMC4105845

[11] Turcan S , Rohle D, Goenka A, et al IDH1 mutation is sufficient to establish the glioma hypermethylator phenotype. Nature. 2012;483:479-483. 10.1038/nature10866 22343889 PMC3351699

[12] Lu C , Ward PS, Kapoor GS, et al IDH mutation impairs histone demethylation and results in a block to cell differentiation. Nature. 2012;483:474-478. 10.1038/nature10860 22343901 PMC3478770

[13] Kitagawa Y , Kobayashi A, Cahill DP, Wakimoto H, Tanaka S. Molecular biology and novel therapeutics for IDH mutant gliomas: the new era of IDH inhibitors. Biochim Biophys Acta Rev Cancer. 2024;1879:189102. 10.1016/j.bbcan.2024.189102 38653436

[14] Tateishi K , Wakimoto H, Iafrate AJ, et al Extreme vulnerability of IDH1 mutant cancers to NAD+ depletion. Cancer Cell. 2015;28:773-784. 10.1016/j.ccell.2015.11.006 26678339 PMC4684594

[15] Núñez FJ , Mendez FM, Kadiyala P, et al IDH1-R132H acts as a tumor suppressor in glioma via epigenetic up-regulation of the DNA damage response. Sci Transl Med. 2019;11:eaaq1427. 10.1126/scitranslmed.aaq1427 30760578 PMC6400220

[16] Kamson DO , Puri S, Sang Y, et al Impact of frontline ivosidenib on volumetric growth patterns in isocitrate dehydrogenase–mutant astrocytic and oligodendroglial tumors. Clin Cancer Res. 2023;29:4863-4869. 10.1158/1078-0432.CCR-23-0585 37382607 PMC10756070

[17] Mellinghoff IK , Lu M, Wen PY, et al Vorasidenib and ivosidenib in IDH1-mutant low-grade glioma: a randomized, perioperative phase 1 trial. Nat Med. 2023;29:615-622. 10.1038/s41591-022-02141-2 36823302 PMC10313524

[18] Rohle D , Popovici-Muller J, Palaskas N, et al An inhibitor of mutant IDH1 delays growth and promotes differentiation of glioma cells. Science (1979). 2013;340:626-630. 10.1126/science.1236062 PMC3985613 23558169

[19] Mellinghoff IK , Van Den Bent MJ, Blumenthal DT, et al Vorasidenib in IDH1- or IDH2-mutant low-grade glioma. N Engl J Med. 2023;389:589-601. 10.1056/NEJMoa2304194 37272516 PMC11445763

[20] Kessler J , Güttler A, Wichmann H, et al IDH1R132H mutation causes a less aggressive phenotype and radiosensitizes human malignant glioma cells independent of the oxygenation status. Radiother Oncol. 2015;116:381-387. 10.1016/j.radonc.2015.08.007 26328938

[21] Li S , Chou AP, Chen W, et al Overexpression of isocitrate dehydrogenase mutant proteins renders glioma cells more sensitive to radiation. Neuro Oncol. 2013;15:57-68. 10.1093/neuonc/nos261 23115158 PMC3534418

[22] Tran AN , Lai A, Li S, et al Increased sensitivity to radiochemotherapy in IDH1 mutant glioblastoma as demonstrated by serial quantitative MR volumetry. Neuro Oncol. 2014;16:414-420. 10.1093/neuonc/not198 24305712 PMC3922511

[23] Buckner JC , Shaw EG, Pugh SL, et al Radiation plus procarbazine, CCNU, and vincristine in low-grade glioma. N Engl J Med. 2016;374:1344-1355. 10.1056/NEJMoa1500925 27050206 PMC5170873

[24] Molenaar RJ , Botman D, Smits MA, et al Radioprotection of *IDH1*-mutated cancer cells by the IDH1-mutant inhibitor AGI-5198. Cancer Res. 2015;75:4790-4802. 10.1158/0008-5472.CAN-14-3603 26363012

[25] Kadiyala P , Carney SV, Gauss JC, et al Inhibition of 2-hydroxyglutarate elicits metabolic reprogramming and mutant IDH1 glioma immunity in mice. J Clin Invest. 2021;131:e139542. 10.1172/JCI139542 33332283 PMC7880418

[26] Nagashima H , Lee CK, Tateishi K, et al Poly(ADP-ribose) glycohydrolase inhibition sequesters NAD+ to potentiate the metabolic lethality of alkylating chemotherapy in IDH-Mutant tumor cells. Cancer Discov. 2020;10:1672-1689. 10.1158/2159-8290.cd-20-0226 32606138 PMC7642007

[27] Wakimoto H , Tanaka S, Curry WT, et al Targetable signaling pathway mutations are associated with malignant phenotype in *IDH*-Mutant ­gliomas. Clin Cancer Res. 2014;20:2898-2909. 10.1158/1078-0432.CCR-13-3052 24714777 PMC4070445

[28] Wakimoto H , Mohapatra G, Kanai R, et al Maintenance of primary tumor phenotype and genotype in glioblastoma stem cells. Neuro Oncol. 2012;14:132-144. 10.1093/neuonc/nor195 22067563 PMC3266381

[29] Wakimoto H , Kesari S, Farrell CJ, et al Human glioblastoma–derived. Cancer stem cells: establishment of invasive glioma models and treatment with oncolytic herpes simplex virus vectors. Cancer Res. 2009;69:3472-3481. 10.1158/0008-5472.CAN-08-3886 19351838 PMC2785462

[30] Amankulor NM , Kim Y, Arora S, et al Mutant IDH1 regulates the tumor-associated immune system in gliomas. Genes Dev. 2017;31:774-786. 10.1101/gad.294991.116 28465358 PMC5435890

[31] Urban DJ , Martinez NJ, Davis MI, et al Assessing inhibitors of mutant isocitrate dehydrogenase using a suite of pre-clinical discovery assays. Sci Rep. 2017;7:12758. 10.1038/s41598-017-12630-x 28986582 PMC5630632

[32] Parsons DW , Jones S, Zhang X, et al An integrated genomic analysis of human glioblastoma multiforme. Science (1979). 2008;321:1807-1812. 10.1126/science.1164382 PMC2820389 18772396

[33] Yan H , Parsons DW, Jin G, et al *IDH1* and *IDH2* mutations in gliomas. N Engl J Med. 2009;360:765-773. 10.1056/NEJMoa0808710 19228619 PMC2820383

[34] Ichimura K , Pearson DM, Kocialkowski S, et al IDH1 mutations are present in the majority of common adult gliomas but rare in primary glioblastomas. Neuro Oncol. 2009;11:341-347. 10.1215/15228517-2009-025 19435942 PMC2743214

[35] Sanson M , Marie Y, Paris S, et al Isocitrate dehydrogenase 1 codon 132 mutation is an important prognostic biomarker in gliomas. J Clin Oncol. 2009;27:4150-4154. 10.1200/JCO.2009.21.9832 19636000

[36] Gravendeel LAM , Kloosterhof NK, Bralten LBC, et al Segregation of non-p.R132H mutations in *IDH1* in distinct molecular subtypes of glioma. Hum Mutat. 2010;31:E1186-E1199. 10.1002/humu.21201 20077503

[37] Kopinja J , Sevilla RS, Levitan D, et al A brain penetrant mutant IDH1 inhibitor provides In vivo survival benefit. Sci Rep. 2017;7:13853. 10.1038/s41598-017-14065-w 29062039 PMC5653818

[38] Bao ZS , Chen HM, Yang MY, et al RNA-seq of 272 gliomas revealed a novel, recurrent *PTPRZ1-MET* fusion transcript in secondary glioblastomas. Genome Res. 2014;24:1765-1773. 10.1101/gr.165126.113 25135958 PMC4216918

[39] Hu H , Mu Q, Bao Z, et al Mutational landscape of secondary glioblastoma guides MET-targeted trial in brain tumor. Cell. 2018;175:1665-1678.e18. 10.1016/j.cell.2018.09.038 30343896

[40] Lal B , Xia S, Abounader R, Laterra J. Targeting the c-met pathway potentiates glioblastoma responses to γ-radiation. Clin Cancer Res. 2005;11:4479-4486. 10.1158/1078-0432.CCR-05-0166 15958633

[41] De Bacco F , Luraghi P, Medico E, et al Induction of MET by ionizing radiation and its role in radioresistance and invasive growth of cancer. J Natl Cancer Inst. 2011;103:645-661. 10.1093/jnci/djr093 21464397

[42] Pardo B , Gómez-González B, Aguilera A. DNA repair in mammalian cells: DNA double-strand break repair: how to fix a broken relationship. Cell Mol Life Sci. 2009;66:1039-1056. 10.1007/s00018-009-8740-3 19153654 PMC11131446

[43] Chowdhury R , Yeoh KK, Tian Y, et al The oncometabolite 2-hydroxyg­lu­tarate inhibits histone lysine demethylases. EMBO Rep. 2011;12:463-469. 10.1038/embor.2011.43 21460794 PMC3090014

[44] Cloughesy TF , van den Bent MJ, Touat M, et al Vorasidenib in IDH1-mutant or IDH2-mutant low-grade glioma (INDIGO): secondary and exploratory endpoints from a randomised, double-blind, placebo-controlled, phase 3 trial. Lancet Oncol. 2025;26:1665-1675. 10.1016/S1470-2045(25)00472-3 41175888

[45] McAbee JH , Degorre-Kerbaul C, Tofilon PJ. In vitro methods for the study of glioblastoma stem-like cell radiosensitivity. Methods Mol Biol. 2021;2269:37-47. 10.1007/978-1-0716-1225-5_3 33687670 PMC10802913

[46] Peterson E , Parsels LA, Parsels JD, et al Inhibition of H3K27M-enhanced ATM signaling increases radiation efficacy in diffuse midline glioma. Cancer Biol. 2024. 10.1101/2024.11.01.621526

[47] Rohle D , Popovici-Muller J, Palaskas N, et al An inhibitor of mutant IDH1 delays growth and promotes differentiation of glioma cells. Science (1979). 2013;340:626-630. 10.1126/science.1236062 PMC3985613 23558169

[48] Konteatis Z , Artin E, Nicolay B, et al Vorasidenib (AG-881): a first-in-class, brain-penetrant dual inhibitor of mutant IDH1 and 2 for treatment of glioma. ACS Med Chem Lett. 2020;11:101-107. 10.1021/acsmedchemlett.9b00509 32071674 PMC7025383

